# Noninvasive real-time assessment of intracranial pressure after traumatic brain injury based on electromagnetic coupling phase sensing technology

**DOI:** 10.1186/s12883-021-02049-3

**Published:** 2021-01-18

**Authors:** Gen Li, Wang Li, Jingbo Chen, Shuanglin Zhao, Zelin Bai, Qi Liu, Qi Liao, Minglian He, Wei Zhuang, Mingsheng Chen, Jian Sun, Yujie Chen

**Affiliations:** 1 Department of Biomedical Engineering, School of Pharmacy and Bioengineering, Chongqing University of Technology, Chongqing, China; 2 Department of Biomedical Engineering, Army Medical University, 30 Gaotanyan Street, Shapingba District, Chongqing, 400038 China; 3 Department of Neurosurgery, Southwest Hospital, Army Medical University, 29 Gaotanyan Street, Shapingba District, Chongqing, 400038 China; 4 State Key Laboratory of Trauma, Burn and Combined Injury, Army Medical University, Chongqing, China; 5 Chongqing Key Laboratory of Precision Neuromedicine and Neuroregenaration, Army Medical University, Chongqing, China

**Keywords:** Electromagnetic coupling phase sensing, Traumatic brain injury, Intracranial pressure, Classification decision algorithm, Noninvasive monitor

## Abstract

**Background:**

To investigate the feasibility of intracranial pressure (ICP) monitoring after traumatic brain injury (TBI) by electromagnetic coupling phase sensing, we established a portable electromagnetic coupling phase shift (ECPS) test system and conducted a comparison with invasive ICP.

**Methods:**

TBI rabbits’ model were all synchronously monitored for 24 h by ECPS testing and invasive ICP. We investigated the abilities of the ECPS to detect targeted ICP by feature extraction and traditional classification decision algorithms.

**Results:**

The ECPS showed an overall downward trend with a variation range of − 13.370 ± 2.245° as ICP rose from 11.450 ± 0.510 mmHg to 38.750 ± 4.064 mmHg, but its change rate gradually declined. It was greater than 1.5°/h during the first 6 h, then decreased to 0.5°/h and finally reached the minimum of 0.14°/h. Nonlinear regression analysis results illustrated that both the ECPS and its change rate decrease with increasing ICP post-TBI. When used as a recognition feature, the ability (area under the receiver operating characteristic curve, AUCs) of the ECPS to detect ICP ≥ 20 mmHg was 0.88 ± 0.01 based on the optimized adaptive boosting model, reaching the advanced level of current noninvasive ICP assessment methods.

**Conclusions:**

The ECPS has the potential to be used for noninvasive continuous monitoring of elevated ICP post-TBI.

**Supplementary Information:**

The online version contains supplementary material available at 10.1186/s12883-021-02049-3.

## Background

Intracranial pressure (ICP) management is of great significance for improving the outcomes of traumatic brain injury (TBI) patients [1]. However, the invasive monitoring of ICP, the gold standard for intracranial hypertension, is not routinely undertaken due to the scarcity of neurosurgeons and contraindications such as thrombocythemia or coagulopathy [2]. In addition, its surgical procedures require specific expertise and exposure to malposition, hemorrhage and infection [3, 4]. Therefore, it is urgent to develop reliable noninvasive methods for monitoring ICP. Electromagnetic coupling phase sensing technology is used to measure the phase differences, namely, the electromagnetic coupling phase shift (ECPS), between the disturbance and excitation fields; this approach has the advantages of noninvasiveness, strong penetrability, and real-time monitoring. Earlier studies reported by Griffiths, Scharfetter, Gonzalez et al. have shown that the ECPS is characterized by complex electrical impedance of tissues [5–7]. Our previous study found that ICP and ECPSs have a negative correlation in a rabbit model of intracranial hemorrhage [8]. Furthermore, 24-h real-time monitoring research of cerebral edema in rabbits verifies that the ECPS can obtain information about brain volume changes that are directly related to ICP [9]. In this study, we established a portable ECPS test system and investigated its feasibility of monitoring ICP post-TBI in rabbits with brain contusion and laceration by evaluating the relationship between changes in the complex electrical impedance of the brain and intracranial hypertension formation.

## Methods

### Detection principle

**Fig. 1:**
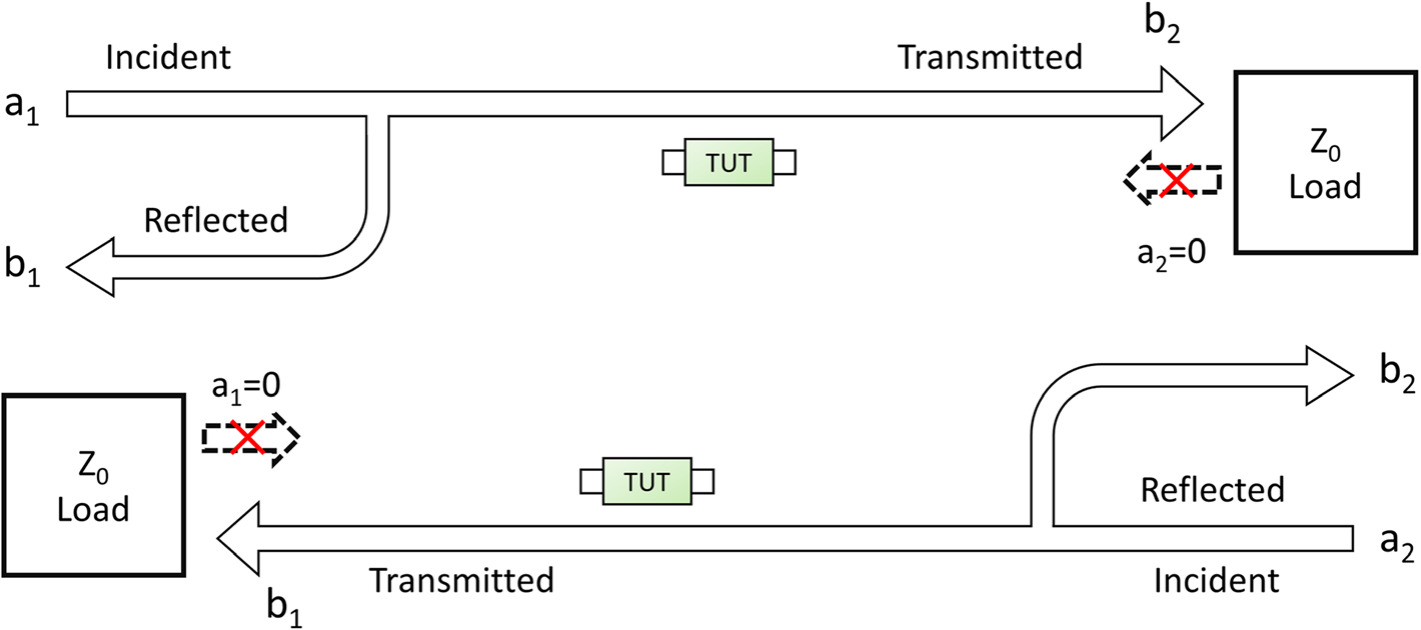
ECPS detection principle based on a two-port test network

For a two-port test system containing a signal source and a target being tested, the input and output relationship can be defined by using incident signals, reflected signals and transmitted signals. As shown in Fig. 1, when Port 1 is added with an incident signal a1 and Port 2 has no input (a2 = 0), some of the incident signal is reflected due to the mismatch of this port; that is, the reflected signal, and the rest of the signals are transmitted to Port 2 through tissues, referred to as the transmitted signal.

The ratio of the transmitted signal to the incident signal is defined as the transmission coefficient T:

1 $$ \mathrm{T} =\frac{V_{Transmitted}}{V_{Incident}}=\tau \angle \varphi $$

where *τ* is the amplitude of the transmission coefficient, whose magnitude is equal to the modulus of T, and the phase of the transmission coefficient *φ* is the change between the phase of the transmission signal and the phase of the original incident signal, namely, the ECPS, whose size is related to the complex electrical impedance of tissues [10]. The electromagnetic properties of the main intracranial tissues, such as cerebrospinal fluid (CSF), cerebral blood flow (CBF), gray matter, white matter, and water, are quite different according to the reported study by C. Gabriel [11]. The ICP increase post-TBI is accompanied by obvious changes in the relative volume of these tissues, which causes variations in the overall complex electrical impedance of the brain. Therefore, the ECPS signal theoretically contains information about elevated ICP.

### ECPS test system

Based on the principle of the two-port test network, we constructed a portable ECPS test system in this study. It was mainly composed of a vector network analyzer (Copper Mountain, M5065), a coil sensor suitable for animal experiments and real-time monitoring software. The vector network analyzer contained the signal source, the signal separation module, the amplitude-phase receiver and the signal processing module, which was responsible for the output of the excitation signal, directional separation of incident and reference signals, independent acquisition of reference and transmitted signals, and amplitude-phase calculation. According to our previous studies, the phase shift between the transmitted signal and reference signal can detect changes in the complex electrical impedance of tissues. The coil sensor included excitation and detection coils; 10 turns of AWG32 copper paint wire were wound at both ends of the plexiglass tube. Based on the skull size of the rabbits, the radius of the coils was the same (R1 = R2 = 5.2 cm), the distance was 10 cm, and a sufficiently large hole was opened in the proper position of the plexiglass tube to facilitate the placement of the ICP fiber detector [12]. The real-time monitoring software on a PC was programmed in the LabVIEW environment, which communicated with a vector network analyzer via USB. It could automatically set measurement parameters, continuously read the real-time ECPS value at the targeted frequency point, adjust the sampling rate and display the dynamic wave of test results.

### ICP and ECPS real-time synchronous monitoring

To investigate the relationship between ICP and the ECPS, we established a rabbit model of TBI by the liquid nitrogen freezing method and carried out real-time synchronous monitoring of ECPS and ICP for 24 h. All animal procedures were approved by the Laboratory Animal Welfare and Ethics Committee of the Army Medical University (AMUWEC2020715), performed in accordance with the guidelines by the National Institutes of Health Guide for the Care and Use of Laboratory Animals, and reported by following the Animals in Research: Reporting In Vivo Experiments (ARRIVE) guidelines. All efforts were made to minimize the suffering of rabbits during experiments.

Thirty-two healthy rabbits (Male, 2.5 kg ± 0.3 kg) were provided by the Animal Center of Amy Medical University and divided into experimental group (*n* = 20) and control group (*n* = 12). For the experimental group, the liquid nitrogen freezing method was used to establish a model of TBI, which can better simulate craniocerebral contusion and laceration; this approach has the advantages of a single injury mechanism, strong repeatability and low mortality, making it convenient for qualitative research [13]. After anesthesia by injection of urethane (25%, 5 ml/kg) through ear margin, the frozen position was determined by the stereotactic locator. The frozen position was 6 mm on the right side of the sagittal seam and 1 mm below the coronal seam. A drill with a diameter of 1 mm was used to drill into the skull to form a bone window with a diameter of 5 mm and a depth of 3.5 mm. Then, a freezing pen soaked in liquid nitrogen for an extended period of time was inserted into the bone window twice to achieve external skull freezing. The freezing temperature was − 196 °C, and the freezing time was 60 s. The skull cavity was sealed with dental cement after freezing. The Camino mpm-1 ICP monitor was selected as a reference for ECPS. To embed the ICP fiber optic sensor, it’s necessary to drill a 2 mm hole with the frozen position axisymmetrical to the sagittal suture. In comparison, the rabbits in the control group endured the same operation as experimental group only without extracranial freezing.

**Fig. 2:**
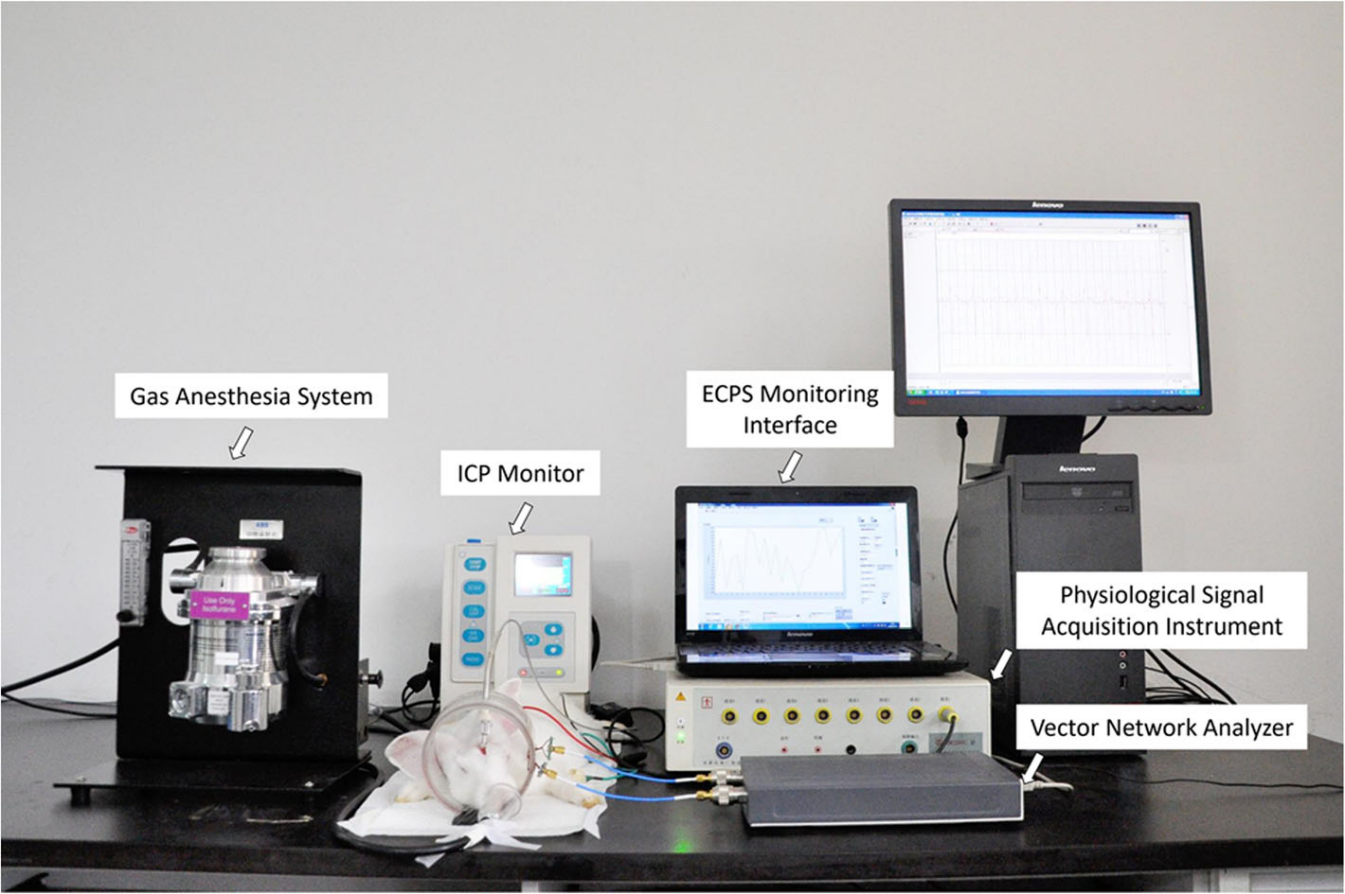
Experimental setup to monitor the ECPS and invasive ICP in rabbits

As shown in Fig. 2, all the rabbits in the experimental group and the control group underwent 24-h real-time synchronous monitoring by the ECPS test system and the ICP monitor after above operations. They were lying on the bench, abdomen facing down, limbs facing out, head in the excitation coil and the detection coil axis offset the lower position, about 2.2 cm. The frequency sweeping from 300 kHz to 500 MHz shows that 64.14396 MHz has the highest transmission coefficient amplitude, and its corresponding reflection coefficient has a low amplitude, which means that impedance matching is optimal and that the ECPS at this frequency point had high sensitivity and stability [14]. Therefore, it was used to detect ECPS by real-time monitoring software. A physiological signal acquisition instrument (RM6280C, Chengdu Instrument Factory, Chengdu, China) was adopted to monitor the heart rate changes in the rabbits. When the respiratory frequency and heart rates of rabbits were normal, the 24-h real-time data of ICP and ECPS were continuously recorded under the same sampling rate (60 times/h). During the monitoring process, rabbits were subjected to continuous anesthesia using 2% isoflurane gas at a flow rate of 0.6 L/min. Meanwhile, respiratory frequency and heart rate amplitude were monitored to ensure the vital sign stability of the rabbits. After experiments, rabbits were euthanized by the air injection method.

### ECPS and ICP signal processing

Through spectrum analysis of the ECPS original signal, it was found that the frequency reflecting the overall trend change was concentrated in the low frequency band [15]. In this study, the wavelet transform method was used to process ECPS signals. The Daubechies wavelet (order 5) with 8 discrete wavelet decomposition layers was selected to decompose the original signal to layers A1-A8 in accordance with the frequency band. The analysis of these 8 layers shows that the components of the high-frequency interference signal are mainly concentrated in A1-A7. Next, the components of the seven layers A1-A7 were removed, the sequence of the remaining A8 layers was restored, and wavelet reconstruction was performed so that most of the interference would be filtered out of the obtained signal. Then, the ECPS signal after filtering and denoising and the ICP were resampled with a sampling interval of 30 min, which was used to analyze the deference of change trend in ICP and ECPS between experimental group and control group more clearly.

### Statistical analysis of the relationship between ICP and ECPS

All data are expressed as the mean ± standard deviation from at least twelve independent experiments. According to the mechanism of intracranial compensation, the change rate of ECPS and ICP was comparatively analyzed during the 24-h monitoring process to determine if the ECPS can reflect the ICP rising at different stages after TBI.

Nonlinear regression was used to investigate the relationship between ICP and ECPS. In Origin 9.1 (Origin Lab, Massachusetts, MA, USA), the mean ECPS from the experimental rabbits was set as independent *variable θ*, the mean ICP of the experimental rabbits was set as dependent variable *P*, and the first-order exponential decay function was selected to perform nonlinear regression analysis.

### Classification algorithm of intracranial hypertension

To investigate the ability of the ECPS to detect ICP, the ECPS and its change rate (δ) were used as features to classify ICP at different levels in this study. The change rate of the ECPS for each sample was calculated using formula (1).

1 $$ {\delta}_{t_i}=\left\{\begin{array}{cc}\frac{E_{t_{i+1}}-{E}_{t_i}}{t_{i+1}-{t}_i},& {t}_i\in \left[0,24\right)\\ {}0,& {t}_i=24\end{array}\right. $$

*E*_*ti*_ represents the ECPS at *t*_*i*_.

The ECPS and δ were standardized by the z-score method as follows:

2 $$ {X_i}^{\ast }=\frac{X_i-\overline{X}}{\sigma } $$

where *X* is the value of the feature (ECPS, *δ*) and *σ* is the standard deviation.

According to the general fluctuation range of ICP after TBI in the clinic, sample labels were determined as follows:

3 $$ \mathrm{labels}=\left\{\begin{array}{cc}1,& ICP\ge k\\ {}0,& ICP<k\end{array}\right.\kern0.5em \mathrm{for}\ k\ \mathrm{in}\ \left[15,25\right] $$

Eleven different datasets were obtained after traversing all values of ICP in this interval. Although the X value in these datasets was the same, the labels were completely different. We investigated the ability of ECPS and δ to recognize ICP distribution based on the classification effect at different labels. Four traditional classification decision algorithms, support vector machine, decision tree, neural network and adaptive boosting, were performed in this study. Twelve of the 20 rabbits in the experimental group were used as the training set, and the rest were used as the test set. During the classification process, the grid search method was adopted to optimize the parameters of the four classification models. Finally, we obtained the best classification model by comparing the accuracy in the four optimized models and plotting the receiver operating characteristics (ROC) curve to investigate its performance for recognizing whether ICP was greater than 20 mmHg in 10 random groupings.

## Results

### Twenty-four-hour monitoring of ECPS and ICP

**Fig. 3:**
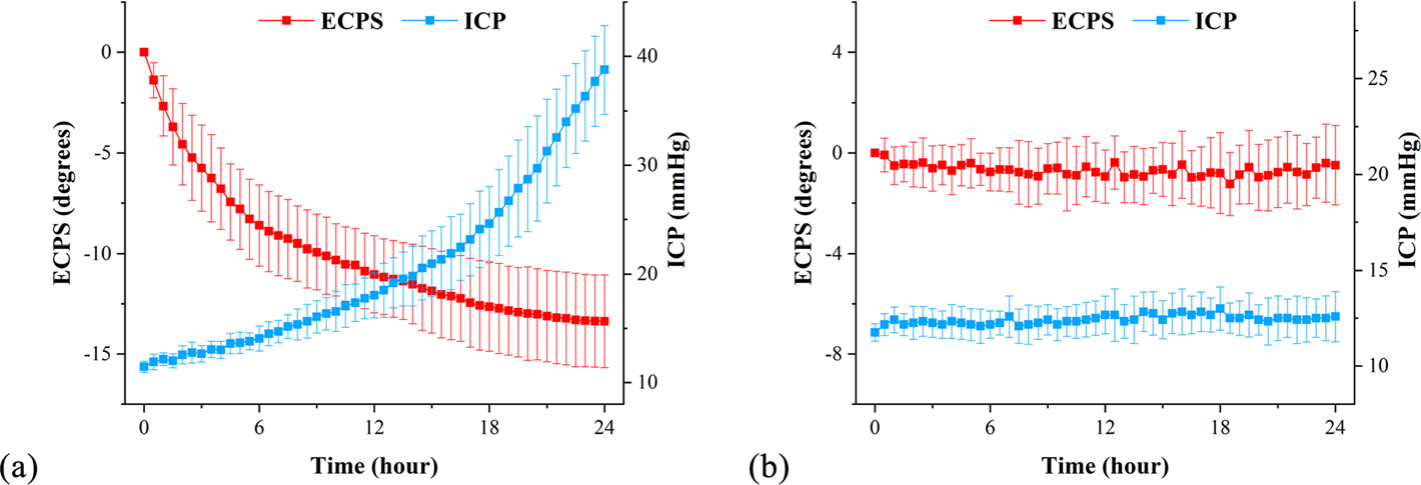
Changes in the ECPS and ICP during 24-h monitoring after traumatic brain injury. (**a**) The mean ± std. changes of the ECPS and ICP in the experimental group (*n* = 20) during the 24-h monitoring period. (**b**) The mean ± std. changes of the ECPS and ICP in the control group (*n* = 12) during the 24-h monitoring period

Figure 3(a) showed the 24-h mean ± standard deviation changes of ECPS and ICP in the experimental group (*n* = 20). In the 24 h after freezing, ECPS showed an overall downward trend with a variation range of − 13.370 ± 2.245°. Meanwhile, the ICP gradually increased from the initial value of 11.450 ± 0.510 mmHg to 38.750 ± 4.064 mmHg. The 24-h mean ± standard deviation changes of ECPS and ICP in the control group (*n* = 12) were shown in Fig. 3(b). The rabbits in the control group underwent the same operation as those in the experimental group only without freezing. The CSF was basically in a normal circulation state and the brain volume did not expanded obviously. Only very weak intravenous capillary bleeding was observed near the probe of the ICP monitoring. ECPS varied from 0° to − 0.489° and ICP fluctuated around 11.75 mmHg within 24 h, which had no changes compared with those in the experimental group. As the freezing method mainly simulates craniocerebral contusion, the decreased ECPS and the increased ICP in the experimental group are caused by changes in intracranial pathophysiological states.

### Relationship between ECPS and ICP post-TBI

**Fig. 4:**
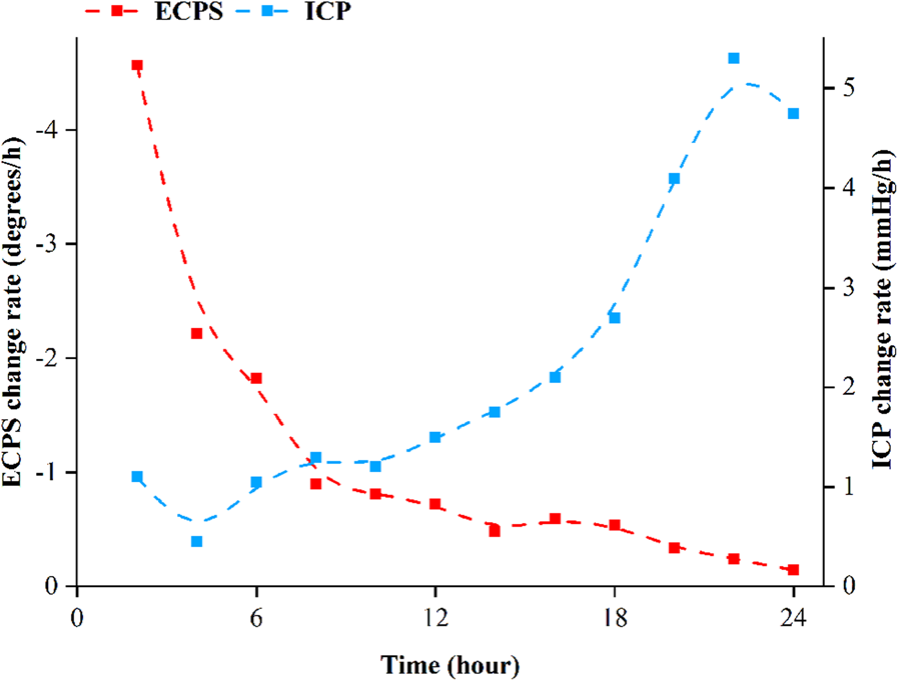
The curve contrast diagram of 24-h average change rates in the ECPS and ICP

Figure 4 shows the change rate of ICP in the experimental group during 24-h monitoring. It fluctuates at approximately 1 mmHg/h during the first 6 h, then gradually increases to 2.7 mmHg/h and rapidly reaches a maximum of 5.3 mmHg/h during the final 6 h. Compared with ICP, the change rate of the ECPS gradually declines with time. The change rate of the ECPS is greater than 1.5°/h during the first 6 h, then decreases to 0.5°/h in the next 12 h and reaches a minimum of 0.14°/h during the final 6 h, which is opposite of the trend observed for ICP. Among intracranial tissues, CSF has the highest electromagnetic property, CBF is the second highest, and brain parenchyma is the lowest. In the CSF compensation period, the volume changes of CSF would cause the ECPS to change rapidly ECPS while ICP remains stable. Afterwards, CBF gradually plays a compensatory role. As its electromagnetic property and compensation ability are weaker than that of CSF, the ECPS changes slowly, and ICP starts to increase significantly. When the intracranial compensatory effect is exhausted and the intracranial space becomes very narrow, variations in the ECPS are not evident, but any slight increase in brain volume would lead to dramatic changes in ICP [16]. Combined with the 24-h monitoring outcomes of the ECPS and invasive ICP, we found that the ECPS and its change rate both decrease with an increase in ICP post-TBI.

**Fig. 5:**
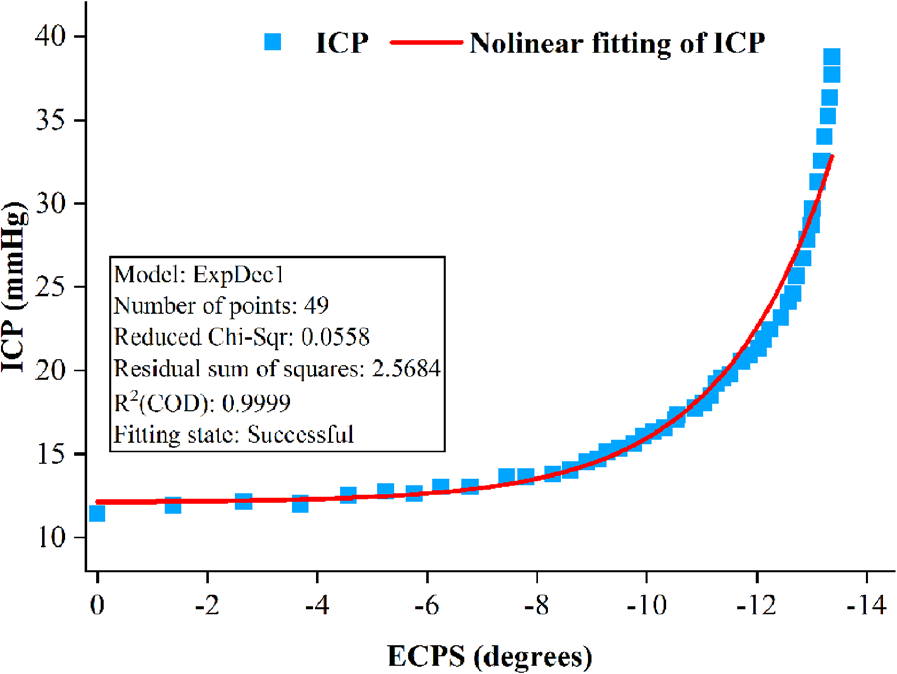
The exponential decay function fitting curve of the ECPS and ICP 24 h after traumatic brain injury

As the ECPS does not have a simple linear correlation with ICP, we used the first-order exponential decay function to conduct nonlinear curve fitting for the data. As shown in Fig. 5, the relative probability value corresponding to the F-statistic is less than 0.05, R^2^ is 0.9999 and the reduced Chi-Sqr is 0.0558, indicating that the data have a significant nonlinear regression relationship and that the fitting effect is very good. According to this function, ICP remains basically unchanged in the CSF compensation period, during which the change rate of ECPS is the largest and then increases exponentially with the exponential decay of ECPS, which strengthens the relationship described above. The detailed distribution of all samples in the experimental group is shown in Supplemental Fig. 1.

### Classification of intracranial hypertension

**Fig. 6:**
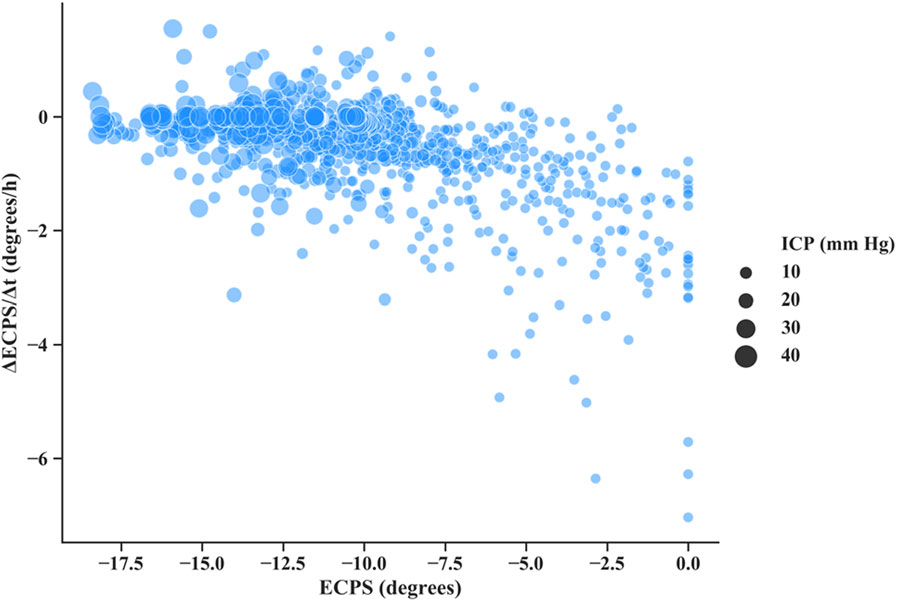
The distribution of the feature space with increasing ICP

According to the relationship between the ECPS and ICP, we regard the ECPS and its change rate (δ) as features to classify different levels of ICP. As shown in Fig. 6, the distribution of the feature space constructed by the ECPS and δ is increasingly congregated with rising ICP. This result illustrates that the two features may have good performance in identifying intracranial hypertension.

**Fig. 7:**
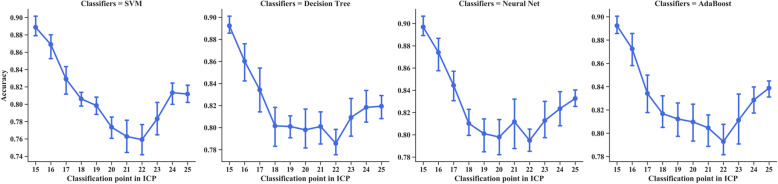
The trend of classification accuracy changes with increasing ICP classification points from 15 mmHg to 25 mmHg

The classification accuracy in the four traditional decision algorithms after parameter optimization is shown in Fig. 7. The classification accuracy of the four models in the ICP label range decreased first and then increased. Among the four models, the accuracy of identifying whether ICP is less than 22 mmHg ranges from 76 to 79% with the worst performance, and the accuracy of identifying whether ICP is less than 15 mmHg ranges from 89 to 90% with the best performance. One reason for these values is that the changes in the ECPS and its change rate have a large difference between the CSF compensation period and the stage when the intracranial compensation is almost lost; the other reason is that there are individual differences among those rabbits. The overall complex electrical impedance of the brain in rabbits with strong intracranial compensatory ability changes more rapidly than that in rabbits with weak intracranial compensatory ability. The optimized parameters and the detailed classification results of the four decision algorithms are shown in Supplemental Table 1. The classification effect and classification boundary in eleven datasets are shown in Supplemental Fig. 2.

For the four different decision algorithms, the adaptive boosting model has the best performance. Its classification accuracy is more than 79% at all classification points and reaches 81% for identifying whether ICP is less than 20 mmHg.

**Fig. 8:**
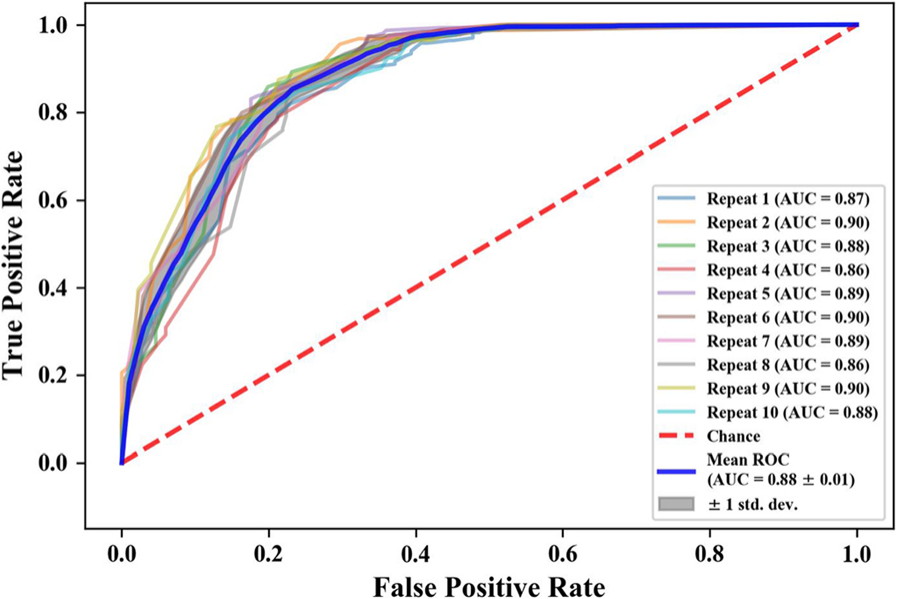
The ROC curve of the ECPS to detect ICP ≥ 20 mmHg

Based on the good performance of the adaptive boosting model, we regarded 20 mmHg as the classification target and plotted its ROC under 10 random groupings. Figure 8 shows that the area under the curve (AUC) is 0.88 ± 0.01 (mean ± std), which indicates that this model has good stability for judging whether ICP is more than 20 mmHg. Therefore, the combination of the two features and adaptive boosting algorithms has good performance in detecting intracranial hypertension.

## Discussion

Targeted treatments for controlling elevated ICP are the key to reducing mortality and disability in TBI patients [17]. Four of the ten treatment methods mentioned in the Guidelines for the Management of Severe Traumatic Brain Injury are related to decompressive craniectomy, hyperosmolar therapy, CSF drainage and pharmacological therapy. Typically, ICP-lowering therapy is initiated when ICP is greater than 20 to 25 mmHg [18]. Based on a total of 459 patients from a database, E. Sorrentino et al. found that the threshold of 22 mmHg for ICP was identified for both survival and favorable outcomes, and this threshold for favorable outcomes was lower (18 mmHg) in females and patients older than 55 years [19]. Single-center multimodal research (*n* = 81) on ICP, arterial blood pressure (ABP) and cerebral perfusion pressure (CPP) monitoring (n = 81) was conducted by V. Petkus et al. demonstrated that age older than 45 years and average ICP above 21.3 mmHg at all monitoring times were associated with unfavorable outcomes in an individual patient [20]. Although some limitations exist, a fixed threshold can serve the role of a successful targeted therapy well. It comprises the advantages of minimum time, knowledge, and computation to make adaptive choices in a real environment.

To date, accurate noninvasive methods to measure ICP have long been sought. Alperin et al. investigated the use of magnetic resonance (MR) as a noninvasive method to evaluate intracranial elastance and pressure in patients [21]. From the pressure-to-volume ratio change, the elastance index was derived from a very good correlation with invasive ICP (R^2^ = 0.965; *P* < 0.005). As it requires a careful selection of representative image slices and the choice of the representative blood vessels, this method cannot be used for continuously monitoring or repeatedly assessing ICP over time. Transcranial Doppler (TCD) is a safe, repeatable and real-time technique to assess ICP and CPP by detecting specific changes in CBF velocity (FV) and pulsatility index (PI). Some reported works demonstrated good performance regarding PI in measuring elevated ICP after TBI [22]. However, Zweifel et al. found a weak correlation between PI and ICP (0.31, *P* = 0.001) in a cohort of 290 patients with TBI, concluding that the value of PI to assess ICP noninvasively is very limited [23]. Flash visual evoked potential (f-VEP) can determine ICP changes by the functional relation between the f-VEP N2 incubation period and the pressure [24]. However, it can only reflect high or moderate ICP changes and is not sensitive to slight ICP changes. As the absorption coefficient of near-infrared light changes in brain tissue after TBI, near-infrared spectroscopy (NIRS) can indirectly monitor ICP. Because the penetrability of near-infrared light on human tissues is very weak, especially the skull, the detection sensitivity of the lesions in the deeper position of the brain is significantly reduced [25]. Bioelectrical impedance technology determines whether the ICP is increased by injecting current into the skull from the skull surface through electrodes and measuring the change in boundary potential [26]. However, in practice, it is difficult to guarantee the electrical contact effect between the electrode and the scalp. In addition, the high resistivity of the skull leads to injection current attenuation and poor penetration, which also seriously affects the measurement accuracy.

The ECPS test system in this study has the advantages of being noninvasive, noncontact and not expensive, having a small size and having strong penetrability, making it more suitable for the bedside management of TBI patients based on ICP monitoring. Compared with the traditional invasive method, it avoids the threat of infection or other complications in patients introduced by the implantation of the ICP probe. It is able to obtain information about intracranial lesions through no direct contact with the human head so as to adapt to some specific situations, such as skin burns and patients with severe skull damage. The users only need place the subject to the demarcate area and run the real-time monitoring software, which does not require complicated professional skills unlike CT, MRI, TCD and et al. As the hardware core of this system, the vector network analyzer (Copper Mountain, M5065) is about 50 times cheaper than that of various imaging equipment. Furthermore, it can be simplified under the condition of ensuring sufficient measurement accuracy to make its cost much lower than 6000 U.S. dollars [27]. The ordinary copper paint wires, the main consumables of this system, are easily available in large quantities.

Owing to the advantages of ECPS, there are a series of studies on diagnosis of cerebrovascular diseases reported by different research groups in the world. Anthony E. Stancombe proposes a portable ECPS imaging system that can capture input signals over a range of approximately 106 dB, which is suitable for detecting realistic brain injuries. It is proved that the system is capable of locating targets with dielectric properties similar to brain tumors and bleeds [28]. The ECPS technology developed by Rubinsky et al. has received U.S. Food and Drug Administration (FDA) approval and is currently used in a variety of clinical studies such as for the development of classifiers to detect edema and hematoma [29], as a tool to study cerebrovascular reactivity [30], or to detect fluid shifts in the brain during dialysis [31]. In 2019, we performed a non-contact and bedside monitoring of cerebral edema in the intensive care unit (ICU) based on ECPS technology, which was approved by Ethics Committee of the First Affiliated Hospital of Southwest Hospital, Chongqing, China. The protocol number of the approval is AF/11/054.0. This research demonstrates the feasibility of ECPS for monitoring the enlargement of brain volume after basal ganglia hemorrhage [16].

In contrast to the normal state, the relative volume of CSF, CBF and brain tissue will change during the pathological process of overall brain volume enlargement. Ljungqvist et al. classified 20 patients with chronic subdural hematomas and 20 healthy volunteers using an electromagnetic coupling device for identifying patients with intracranial hematomas in the prehospital setting [32]. This study was approved by the Regional Ethical Review Board at the University of Gothenburg, Sweden, and reviewed by The Medical Products Agency - Sweden. The results showed that the specificity was 75% at 100% sensitivity. Kellner et al. used a volumetric impedance phase shift spectroscopy (VIPS) device to measure 248 subjects, including healthy volunteers, patients with acute stroke and patients with a wide variety of brain pathologies, which was approved by the institutional review board of each institution, and consent was obtained for all study subjects [33]. It was suggested that the electromagnetic coupling method can detect severe stroke, including emergent large vessel occlusion, and has the potential to improve the triage of patients suffering severe stroke. Chen Mingsheng et al. proved the quantitative relationship between the variation range of ECPS and the volume change in CSF and CBF in the rabbit cerebral hemorrhage model combining the MRI images, which suggested that the changes in ECPS after TBI contain the information of increased ICP [34]. The ECPS signals of the measured objects contain a large amount of noise due to different degrees of body movement caused by different anesthesia depths. After signal processing, most of the noise interference is filtered out, and the ECPS signal that reflects only the changes in the overall complex electrical impedance of the brain is obtained.

The repeatability and consistency of the freezing method simulating brain contusion and laceration is excellent, which is very suitable for qualitative analysis of rising ICP post-TBI. According to different locations of injury, there are two different methods, epidural and extracranial freezing, for the preparation of the TBI model [35]. Epidural freezing can cause a greater degree of damage to the brain and form elevated ICP more rapidly. However, it requires craniotomy, and its operation is more complex, which has an increased risk of massive bleeding. Compared with this procedure, extracranial freezing surgery is very simple, does not require craniotomy and causes no bleeding. The level of brain damage is lower, and the speed of the ICP increase is slower. In clinical practice, patients with TBI are regularly treated by CSF drainage or dehydration to prevent a large increase in ICP. Therefore, extracranial freezing was adopted to cause TBI and form intracranial hypertension in this study. The occurrence of bleeding was avoided as much as possible; only minor bleeding was caused during the insertion of the ICP detection probe, and no intervention treatment was carried out after freezing. Some reported studies have shown that intracranial hemorrhage can also cause changes in the ECPS [36–38]. It is related to changes in the composition and volume of intracranial contents, which is more complicated than ICP. In our previous works, it is proved that ECPS and ICP are negatively correlated in cerebral hemorrhage and traumatic cerebral edema [8, 9, 12]. Based on these, ECPS has the potential to warn intracranial hypertension in a pathological period when the relative volume of intracranial component contents significantly and the ICP does not increase.

From the 24-h synchronous monitoring results, we found that with the increase in ICP after TBI, the absolute change in ECPS gradually increases, and its change rate gradually decreases. The process of TBI-induced ICP elevation can be divided into a CSF compensation period, CBF compensation period and decompensation. Due to weak changes in the CSF compensation period, the current ICP monitoring method is not commonly used in this period [39]. Although we did not conduct a parallel MRI scan to accurately distinguish the three different periods in this study, the ECPS did show high sensitivity when there were no obvious changes in invasive ICP. It is expected to become a referenced supplement for traditional ICP monitoring methods. Regarding the 24-h monitoring, the change rate of ECPS shows a first fast then slow trend. In other words, the sensitivity of the ECPS tends to decay gradually during the pathological process of a TBI-induced rise in ICP. Previous reported work by Kasprowicz M. et al. indicated an exponential relationship between an ICP rise and brain volume expansion [40]. Therefore, it is reasonable that ICP increased exponentially with the exponential decay of the ECPS post-TBI. The rapid decay of the ECPS post-TBI indicates the risk of intracranial hypertension and prompts clinical doctors to intervene as early as possible, which is of great significance for improving treatment conditions and prognostic outcomes.

Based on traditional classification decision algorithms, the ECPS has good performance in detecting intracranial hypertension. The ECPS indirectly reflects the increase in ICP by detecting changes in the overall complex electrical impedance of the brain and contains abundant intracranial pathophysiological information. Therefore, even the same model has some differences in accuracy under different ICP classification points. Based on the optimized integrated learning algorithm, we investigated performance with respect to the ability to recognize whether ICP was greater than 20 mmHg and plotted the ROC. Published studies show that the prediction abilities (AUCs) of TCD for detecting ICP ≥20 mmHg range from 0.62 to 0.92 [41]. A recent systematic review and meta-analysis demonstrated a robust prediction ability (AUCs = 0.94) of optic nerve sheath diameter (ONSD) ultrasonography to detect intracranial hypertension [42]. To evaluate whether retinal measurements from optical coherence tomography (OCT) can serve as an effective surrogate for invasive ICP measurements, J.W. Swanson et al. conducted a cross-sectional study on 79 patients [43]. They found that the OCT parameter had a high AUC ROC curve for detecting elevated ICP (ranging from 0.81–0.86), showing promise as a surrogate, noninvasive measure of ICP. The ability (AUC) of the ECPS to detect ICP ≥ 20 mmHg was 0.88 ± 0.01 in this study, reaching a high level among the noninvasive assessments of ICP mentioned above. Moreover, they may have better performance under other ICP classification points according to the trend of changing accuracy.

The present study has several limitations. The two coils with the same axis in parallel were selected to ensure the universality of the ECPS method itself to measure ICP, as it is difficult to obtain high sensitivity in practical critical care. The injury mechanism of the freezing method is relatively simple. Considering that there are multiple pathogenic factors for TBI-induced intracranial hypertension, a comparative analysis among multiple models, such as free-fall hitting, should be adopted in the future. The change in the characteristics of the ECPS have not been analyzed based on dividing the CSF compensation period, CBF compensation period and decompensation period accurately, as this approach may lead to the loss of some features related to elevated ICP. Although we evaluated the ICP classification points from 15 mmHg to 25 mmHg, the threshold for ICP of rabbits is quite different from that of humans.

## Conclusions

This experimental study proves that the ECPS has the potential for noninvasive monitoring of elevated ICP post-TBI. The ECPS signal is characterized by the complex electrical impedance of the brain, which contains information on intracranial pathophysiological activities. To investigate the feasibility of monitoring ICP by ECPS, we conducted a comparative analysis between ECPS and traditional invasive ICP in rabbits. Both the ECPS and its change rate decreased with the increase in ICP post-TBI during the 24-h monitoring period after TBI, which is consistent with the intracranial compensation-induced changes in the complex electrical impedance of the brain. When the ECPS and its change rate are used as features, the ECPS detects intracranial hypertension well under traditional classification decision algorithms. Based on the optimized adaptive boosting algorithm, ECPS can detect ICP ≥ 20 mmHg with a high specificity.

## Supplementary Information

**Additional file 1: Supplemental Fig. 1.** The detailed distribution of all samples in the experimental group.

**Additional file 2: Supplemental Fig. 2.** The classification effect and classification boundary in eleven datasets.

**Additional file 3: Supplemental Table 1.** The optimized parameters and the detail classification result of the four decision algorithms

## Data Availability

All data generated or analysed during this study are included in this published article and its supplementary information files. Additional personalized data about the current study is available from the corresponding author on reasonable request.

## Abbreviations

ICP: intracranial pressure
TBI: traumatic brain injury
ECPS: electromagnetic coupling phase shift
AUC: Area Under Curve
CSF: cerebrospinal fluid
CBF: cerebral blood flow
ARRIVE: Animals in Research: Reporting In Vivo Experiments
ABP: arterial blood pressure
CPP: cerebral perfusion pressure
MR: magnetic resonance
TCD: Transcranial Doppler
FV: cerebral blood flow velocity
PI: pulsatility index
f-VEP: flash visual evoked potential
NIRS: near-infrared spectroscopy
VIPS: volumetric impedance phase shift spectroscopy
ONSD: optic nerve sheath diameter
OCT: optical coherence tomography
