# Supplementary figures and images for: Noninvasive real-time assessment of intracranial pressure after traumatic brain injury based on electromagnetic coupling phase sensing technology
Source: BMC Neurol. 2021 Jan 18;21:26. doi: 10.1186/s12883-021-02049-3 (PMC7812649; doi:10.1186/s12883-021-02049-3)

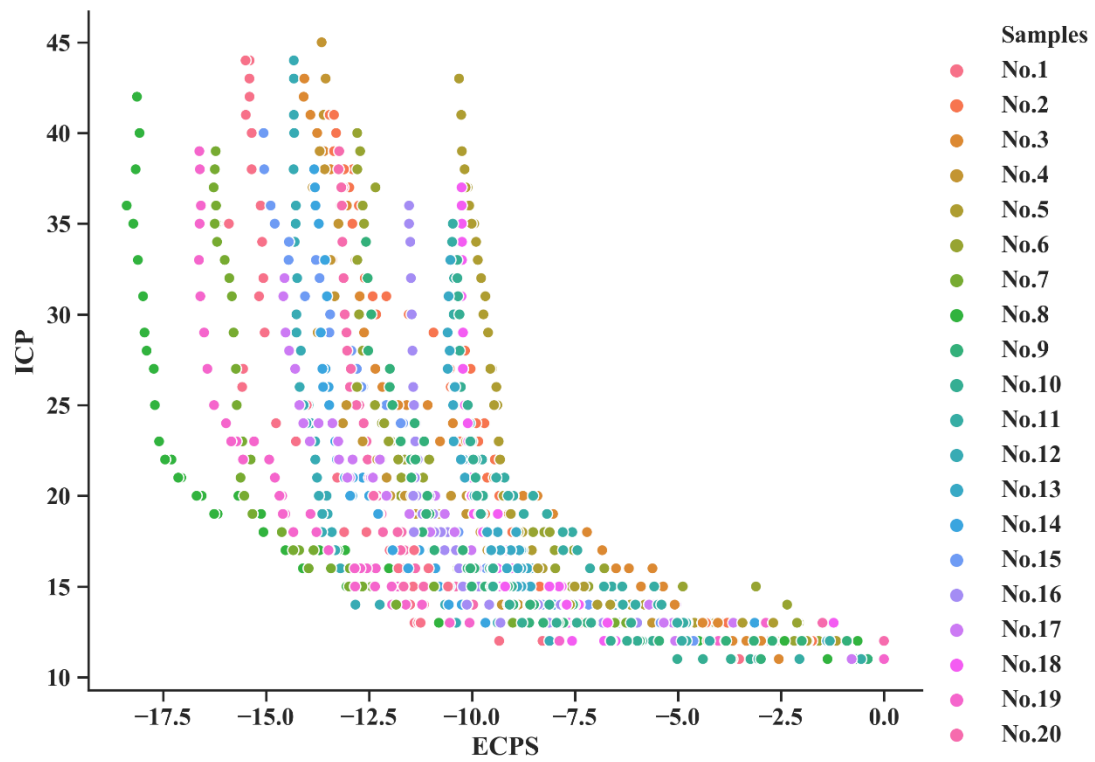

**Additional Figure 1.** The detailed distribution of all samples in the experimental group.

Supplement: Supplementary file 1 — Additional file 1: Supplemental Fig. 1. The detailed distribution of all samples in the experimental group. [file 12883_2021_2049_MOESM1_ESM.pdf]
