# Supplementary material for: Noninvasive real-time assessment of intracranial pressure after traumatic brain injury based on electromagnetic coupling phase sensing technology
Source: BMC Neurol. 2021 Jan 18;21:26. doi: 10.1186/s12883-021-02049-3 (PMC7812649; doi:10.1186/s12883-021-02049-3)
Supplement: Supplementary file 2 — Additional file 2: Supplemental Fig. 2. The classification effect and classification boundary in eleven datasets. [file 12883_2021_2049_MOESM2_ESM.pdf]

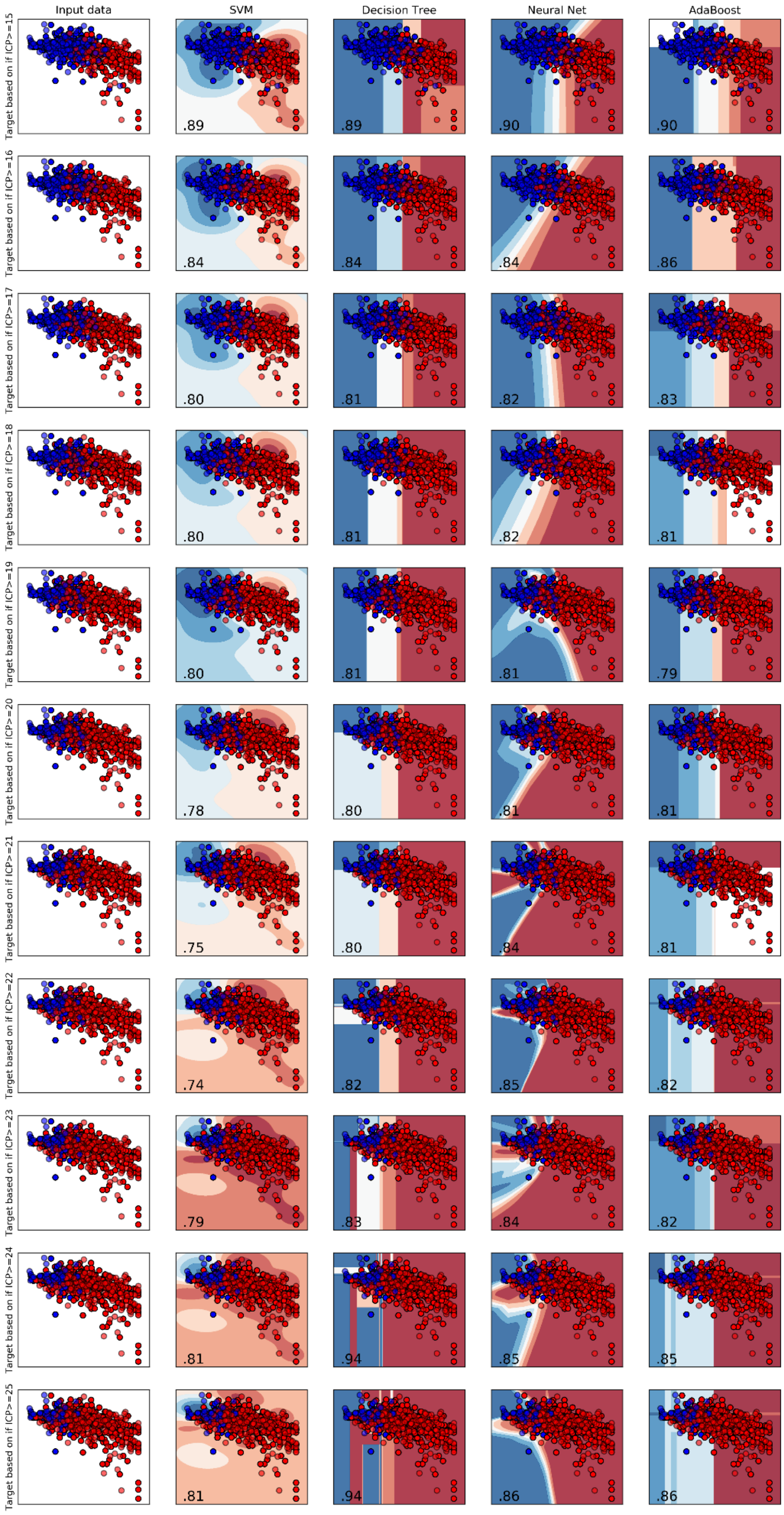

Additional Figure 2. The classification effect and classification boundary in eleven datasets.
